# Pathogen and Hemocyte Dynamics in Three Apiaries Across a Bee Season

**DOI:** 10.1155/tbed/5065424

**Published:** 2025-12-19

**Authors:** Cato Van Herzele, Sieglinde Coppens, Sebastiaan Theuns, Lina De Smet, Jolien Van Cleemput, Dirk C. de Graaf, Hans Nauwynck

**Affiliations:** ^1^ Department of Translational Physiology, Infectiology and Public Health, Ghent University, Merelbeke, Belgium, ugent.be; ^2^ Department of Biochemistry and Microbiology, Ghent University, Ghent, Belgium, ugent.be; ^3^ PathoSense bv, Pastoriestraat 10 2500 Lier, Belgium

## Abstract

Honey bees are dying due to a disease complex consisting of viruses, parasites, chemicals, nutritional deficiencies, and management problems. In the present study, pathogens and hemocytes were analyzed in honey bee hemolymph samples using third‐generation sequencing and flow cytometry in three apiaries over a honey bee season. Using nanopore sequencing, several viruses and bacteria were identified, including the first reported presence in European honey bees of La Jolla virus and Apis rhabdovirus 5. Seasonal pathogen peaks were found, with spring and summer showing the highest loads which coincide with the main flowering periods. Notably, viral infections often did not persist after initial detection despite the continuous appearance of new bees, hinting at colony‐wide transgenerational immunity. However, Hubei partiti‐like virus 34 remained endemic in one apiary and was found even in young bees, raising concerns about its ability to evade the transgenerational immunity. Additionally, dynamics in total hemocyte counts (THCs) were identified. Understanding pathogen and immune factor dynamics throughout the bee season will help identify weak colonies and provide valuable insights into colony collapse and its prevention.

## 1. Introduction

Honey bees are vital contributors to global ecosystems, playing a crucial role in pollinating crops and wild plants which sustain human agriculture and biodiversity. In August 2024, the EU Nature Restoration Law was launched to promote the restoration of ecosystems worldwide, benefiting people, the climate, and the planet. This initiative underscores the urgent need for research and action to safeguard pollinators like honey bees, which are integral to the health of agricultural and natural systems. Honey bees are threatened by a disease complex caused by parasites, viruses, pesticides, nutritional deficiencies, and management problems [1–3]. Some of these pathogens have the capacity to breach the immune barriers and enter the hemocoel, causing a systemic infection or providing an entry portal for other pathogens. These immune barriers are the exoskeleton, the gut barrier, and the cellular and humoral innate immunity. The exoskeleton can be penetrated by *Varroa destructor* mites, which feed on honey bee fat body, providing a perfect entry portal for various viruses and bacteria [4, 5]. The gut barrier can be weakened by *Nosema* sp., which multiply in the epithelial cells of the midgut [6]. Humoral immunity involves soluble effector molecules like antimicrobial peptides, whereas cellular immunity relies on hemocytes, which perform functions such as phagocytosis [7]. Compared to humoral immunity, cellular immunity remains less explored. These hemocytes play a crucial role in controlling systemic infections, though sometimes they can also facilitate them [8].

Various categories of infections affect honey bees. There are overt infections, characterized by noticeable symptoms, which can manifest as either acute or chronic. Covert infections, on the other hand, persistently exist or are latent within an apiary across generations (presumably due to vertical transmission) without obvious symptoms. Since cell cultures are not well established in honey bees, it is unclear if a virus exhibits latency and/or persistence [9]. Technological advancements have made it possible to identify subtle symptoms, complicating the definition of what constitutes a symptom. For instance, Deformed wing virus was traditionally associated with both overt and covert infections, with overt infections causing wing deformities. Covert infections can have detrimental effects on honey bee foraging and long‐term survival [10, 11]. Therefore, the distinction between symptomatic and asymptomatic infections is not always straightforward [12].

In this study, we followed three apiaries during one honey bee season. We focused on systemic viral and bacterial infections using third‐generation nanopore sequencing of honey bee hemolymph of 1‐day‐old bees, foragers, and the frequently overlooked winter bees. In addition, we examined the presence of *Varroa* mites and *Nosema* sp. We also followed the honey bee hemocytes which should combat these systemic infections. This study provides valuable insights into honey bee systemic infection patterns and hemocyte loads dependent on age and season.

## 2. Materials and Methods

### 2.1. Honey Bee Collection and Clinical Observations

A schematic overview of the collected honey bees and hive treatments can be found in Figure 1. Three apiaries, located in Belgium (Flanders), were followed from March 2023 to February 2024. Apiary A comprised a total of eight hives. In contrast, at apiaries B and C, hives were regularly bought and sold, resulting in approximately 25 hives on apiary B and 50 hives on apiary C during the study period. All apiaries housed *Apis mellifera* carnica. At every visit, three 1‐day‐old bees (also referred to as nurses for simplicity) and three foragers per hive or six winter bees were collected per hive, of four hives in total. Additionally, a clinical inspection was performed. If colonies collapsed, dead bees or the remaining alive diseased bees were collected. Taking live diseased bees was preferred if possible as certain viruses can degrade or pathogens can be overgrown by opportunistic bacteria. In March, only 1‐day‐old bees and foragers of apiary B were sampled once. In April, nurses and foragers were sampled once in all apiaries except for foragers in apiary A which were sampled twice. During the honey bee production season, 1‐day‐old bee and forager samples were taken biweekly in May and June. In July, September, and October (only for apiary B as temperatures were still mild) nurse and forager samples were taken monthly. From the end of October (apiary A and C) until February, only winter bees were sampled. These samplings took place once at the end of October (apiary A and C), once in December, and once in February. This was accomplished through the top food entrance or via the hive entrance, thus avoiding the need to open the hive. Samples were not collected in November and January to avoid disturbing the winter cluster. Different interventions were done on the apiaries. On apiary A, one hive swarmed, and this swarm was caught and placed in an empty hive on the apiary in the beginning of June. In June one queen perished, and two other queens were killed and replaced by new queens. In August, one of the new queens died so the hive was merged with the previous mentioned swarm and its queen. Apiary A underwent two *Varroa* mite treatments: the first in summer, using a combination of brood interruption followed by oxalic acid application on August 1, 2023; and the second in winter, using only oxalic acid on February 2, 2024. On apiary B, frequent oxalic acid treatments were performed amongst which one in October with a different composition compared to the other treatments as part of a different study (confidential). On apiary C, a total brood removal of one of the hives was performed to control swarming. Apiary C was treated for *Varroa* mites by taking away all the brood and treating with oxalic acid (August 5, 2023). The specific sampling days can be found in Supporting Information 1: File 1.

Figure 1Overview of apiary interventions and symptoms of potential disease. The bars represent a 2‐week period. This study included 3 apiaries, labeled (A–C).

**Figure:**
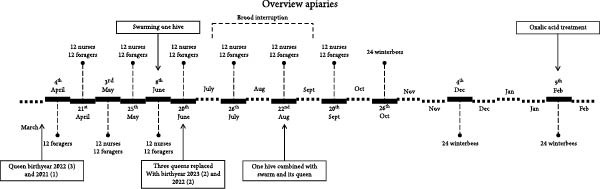
(A)

**Figure:**
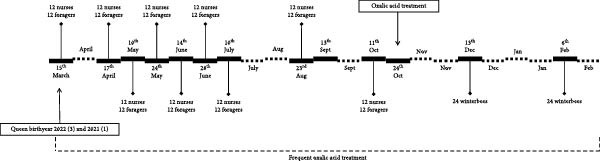
(B)

**Figure:**
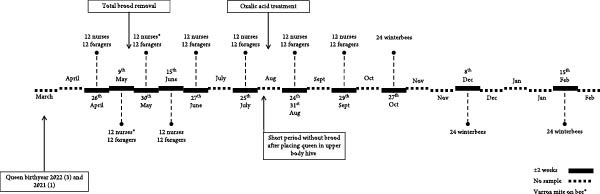
(C)

### 2.2. Hemolymph Collection

Hemolymph collection was performed as reported before [13]. Some small adaptations were made. In short, the bees were sedated on ice for approximately 30 min and surface sterilized using 1/1 hydrogen peroxide (33%) and bleach (15%) followed by a sterile ultrapure (UP) water wash. After drying, using a sterile cotton pad, the bees were flushed with a sterile anticoagulant. The abdomen was punctured between the second and third tergit with a 24G needle. A sterile anticoagulant (0.5 mL, 54 mM EDTA‐PBS, autoclaved) was injected into the thorax using a 30G needle mounted on a 0.5 mL syringe. The anticoagulant hemolymph mixture that exited the bee through the previously made puncture wound, was immediately diluted in 200 µL cold, sterile special bee phosphate‐buffered saline (bPBS) (550 mOsm/L PBS) resulting in 500 µL of hemolymph suspension per bee. The samples were visually inspected for contamination of the gut content. Three aliquots were made for: (1) pathogen analysis (100 µL), (2) hemocyte analysis (300 µL), and (3) cytospin (100 µL). (1) The pathogen analysis was performed using third‐generation nanopore sequencing as reported before [13]. Nanopore metagenomics sequencing was conducted at the PathoSense laboratory, following previously established protocols on 50 µL hemolymph [14–16]. The remaining 50 µL per bee was kept in storage as back up. (2) The second aliquot, which contained 300 µL of hemolymph suspension, was centrifuged at 250 *g* for 10 min (4°C) and resuspended in 200 µL bPBS for flow cytometry (Beckman Coulter) analysis. The flow rate was measured to allow absolute cell counts and the mean was calculated per age, per sampling date, per apiary. The hemocyte count was converted to the total hemocyte count (THC) which is the absolute counts for the original 500 µL hemolymph‐bPBS mixture [17]. (3) The last aliquot was analyzed via cytospin to evaluate the hemolymph samples for contamination. The 100 µL was cytospinned at 600 rpm for 5 min and stained using a May‐Grünwald Giemsa staining according to Van Steenkiste [18].

### 2.3. *Nosema* sp. Analysis Using a Bürker Counting Chamber and (q)PCR

For each age group, the abdomens of two out of three bees or four out of six winter bees were pooled (8 or 16 bees per pool), per apiary and per sampling event, for analysis of *Nosema* sp. DNA extraction followed a protocol developed by the Scientia Terrae Research Institute. To each pooled sample, 4 mL of PBS, glass beads (equivalent volume of 75 µL water), and three metal beads were added. The samples were mechanically homogenized and subsequently an aliquot was diluted with PBS to a total volume of 400 µL, corresponding to a concentration of one bee per mL. Two aliquots were prepared: one (10 µL) was used for quantification with a Bürker counting chamber, and the other (150 µL) was used for PCR and qPCR analysis. The remaining homogenate was stored at −20°C. For spore quantification using the Bürker counting chamber, five squares (with each a dimension of 1 mm × 1 mm × 0.1 mm) on each side of the chamber were counted. If the spore density was too high to allow accurate counting, the sample was further diluted 10‐ or 100‐fold. The number of spores per individual bee was then calculated accordingly [19, 20]. For the PCR and qPCR DNA extraction, 300 µL of lysis buffer (2.5M LiCl; 50 mM Tris‐HCl, pH 8.0; 4% Triton X‐100; 62.5 mM Na_2_‐EDTA, pH 8.0), 450 µL of phenol/chloroform/isoamylalcohol, 75 µL of fine glass beads, and three metal beads were added to the 150 µL aliquot. This mixture was homogenized in a PowerLyzer 24 (Qiagen) at 3500 rpm for 45 s and centrifuged at 10,000 rpm for 5 min to pellet the proteins and cell debris. The supernatant (~400 µL) was transferred to a new tube, and two volumes of ice‐cold 100% ethanol (stored at −20°C) were added. Following incubation at −20°C for at least 15 min, the sample was centrifuged at 10,000 rpm for 5 min, and the supernatant was discarded. The resulting pellet was washed with approximately 300 µL of 70% ethanol (stored at −20°C), centrifuged again for 5 min at 10,000 rpm, air dried, and resuspended in 100 µL of 10 mM Tris‐HCl (pH 8.0). The resuspended DNA (gDNA) was gently mixed and stored at −20°C. PCR was performed in a total reaction volume of 25 µL containing forward primer adapted NOS‐FOR (5′‐TAT GCC GAC GAT GTG ATA TG‐3′) [21] and reverse primer NOS‐REV (5′‐CAC AGC ATC CAT TGA AAA CG‐3′) [22], each at 0.5 µL from 100 µM stock (Integrated DNA Technologies), 2.5 µL of 10 × CoralLoad PCR Buffer (Qiagen), 2 µL of MgCl_2_ (25 mM, Qiagen), 0.5 µL of dNTPs (20 mM, Invitrogen), 0.125 µL of HotStarTaq Plus DNA Polymerase (Qiagen), 18.875 µL of nuclease‐free water, and 1 µL of gDNA. PCR reactions were carried out on a T100 Thermal Cycler (Bio‐Rad Laboratories) with the following program: initial denaturation at 95°C for 5 min; 35 cycles of denaturation at 94°C for 30 s, annealing at 60°C for 30 s, and extension at 72°C for 30 s; followed by a final extension at 72°C for 3 min. PCR products were separated on a 2% agarose gel prepared with 6 g UltraPure Agarose (Invitrogen) in 300 mL Tris‐borate‐EDTA buffer. Positive samples were subsequently screened for *Nosema ceranae* using qPCR. Plasmid DNA was serially diluted (10‐fold, five steps) to generate a standard curve. The primers (forward QNoUF2:5′‐GGA TTG TGC GGC TTA ATT TGA‐3′; reverse QNoCR: 5′‐ACC ACT ATT ATC ATT CTC AAA C‐3′) were dissolved (100 µM) [23]. gDNA was diluted 1:100 with molecular‐grade water and vortexed. Each 15 µL qPCR reaction contained 0.03 µL of each primer, 7.5 µL of 2× Platinum SYBR Green qPCR SuperMix‐UDG, 6.44 µL of nuclease‐free water, and 1 µL of gDNA. Amplification and quantification were conducted with the following cycling conditions: initial denaturation at 95°C for 2 min, followed by 40 cycles of 95°C for 15 s denaturation, 58°C for 20 s primer annealing, and 72°C for 15 s extension, ending with a final extension at 65°C for 5 s. A melt curve analysis was performed immediately afterward to confirm specificity of the amplicon, using a 0.5°C increment every 5 s from 65 to 95°C. The integrity of DNA samples was verified by amplification of the actin gene.

### 2.4. Natural Mite Fall

The free falling *Varroa* mites were counted. For apiary A and C, a paper sheet was put underneath the hive for 1 day. For apiary B, the mites were counted weekly. The mean *Varroa* mite count per day of the four hives was calculated per apiary. For apiary B, the mean of the sampling week was used to calculate the mean *Varroa* mite count per day [24].

### 2.5. Statistical Analysis

All statistical analyses were performed using IBM SPSS Statistics for Windows, Version 29 (IBM Corp., Armonk, NY, USA). First, analyses were conducted separately for each apiary and diseased bees were not included. To evaluate the effect of age on THC concentrations, a blocked analysis of variance (ANOVA) was employed, with hive included as a blocking factor. Post hoc comparisons were adjusted using the Bonferroni correction. Subsequently, within each age group, the effect of Sampling Date was examined using the same blocked ANOVA approach. The comparison of the THC of diseased bees and healthy bees was done using an independent samples *t*‐test. Differences in THC concentrations between apiaries were then assessed within each age group. Again, diseased bees were not included. Prior to analysis, THC data were log‐transformed to improve normality and stabilize variances. Outliers were identified using standardized *Z*‐scores (|Z| > 3). Assumptions of normality and homogeneity of variances were assessed using the Shapiro–Wilk test and Levene’s test, respectively.

## 3. Results

### 3.1. Clinical Observations

On apiary A, no clinical signs or colony mortality were observed. On apiary B, many dead bees were laying in front of one of the four hives included in this study after oxalic acid treatment in October (October 24, 2023). Six dead foragers were collected for pathogen analysis. The dead bees of apiary B were not analyzed for *Nosema* sp. On apiary C, potential disease symptoms were observed. During the second sampling in May, one of the included hives in apiary C showed a patchy brood pattern. In October (October 27, 2023), motionless bees were noticed at the hive entrance of several hives that were initially not included in our study. Eighteen bees that were still alive were collected for analysis in addition to the winter bees of the original four hives. Six were used for pathogen analyses, from which three were used for flow cytometry profiling. The remaining 12 were used for *Nosema* sp. analysis. In December (December 08, 2023), three hives on apiary C died and six bees were sampled for pathogen analysis and 24 dead bees for *Nosema* sp. analysis. In February, a mortality percentage of approximately 27.5% was seen on apiary C including four mating hives and one colony which was placed there recently (during winter). The original four hives that were included in this study survived.

### 3.2. Pathogen Analysis Using Third‐Generation Nanopore Sequencing

A variety of viruses and bacteria were detected. Only the honey bee‐associated bacteria and viruses, as presented in Figure 2 (A–C), will be discussed. Viruses and bacteria, associated with plants and their pests, such as Soybean thrips iflavirus 4, or the kitome, such as *Acinetobacter* sp., were excluded. In total 16 viruses and nine bacterial species were identified. Seven viruses and six bacterial species were detected in apiary A, seven viruses and eight bacterial species in apiary B and 13 viruses and eight bacterial species in apiary C. The raw read sequencing output is uploaded to Sequence Read Archive (PRJNA1285611). The obtained full viral genomes are uploaded on the National Center for Biotechnology Information databank, the accession number can be found in Supporting Information 2: File 2.

Figure 2Third‐generation nanopore sequencing output of 1‐day‐old bees, foragers, dead foragers (Oct^†^, B), live winter bees (Oct [A and C], Dec, and Feb), sick winter bees (Oct^(†)^, C) and dead winter bees (Dec^†^, C). A semi‐quantitative report was generated by comparing the data to the spike‐in virus previously included, enabling categorization of viral and bacterial loads into five levels: very low, low, medium, high, and very high.  ^∗^ Indicate pollen presence in sample.

**Figure:**
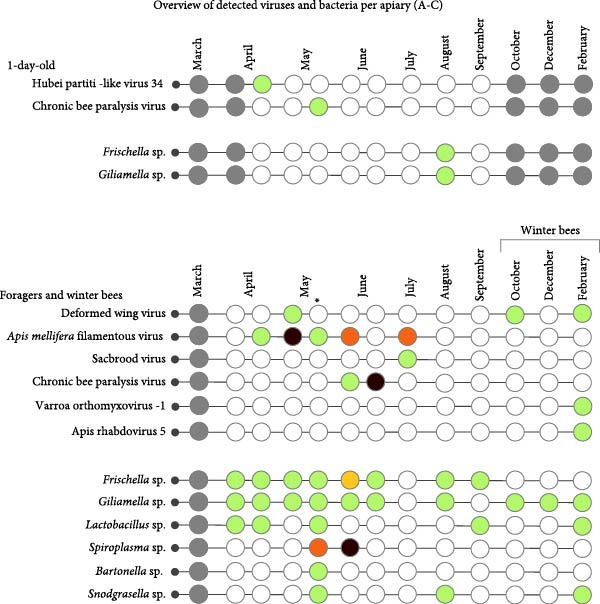
(A)

**Figure:**
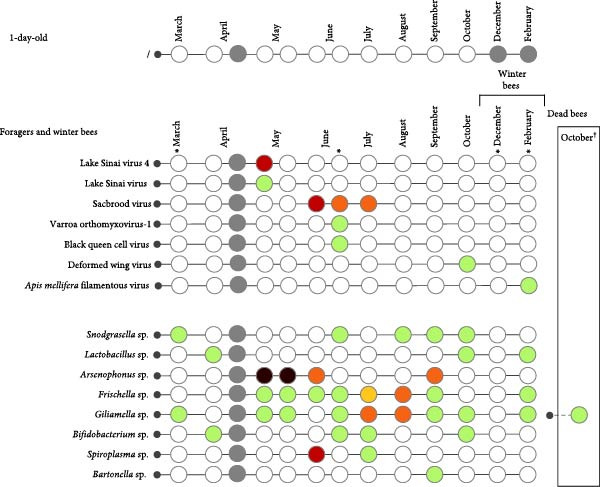
(B)

**Figure:**
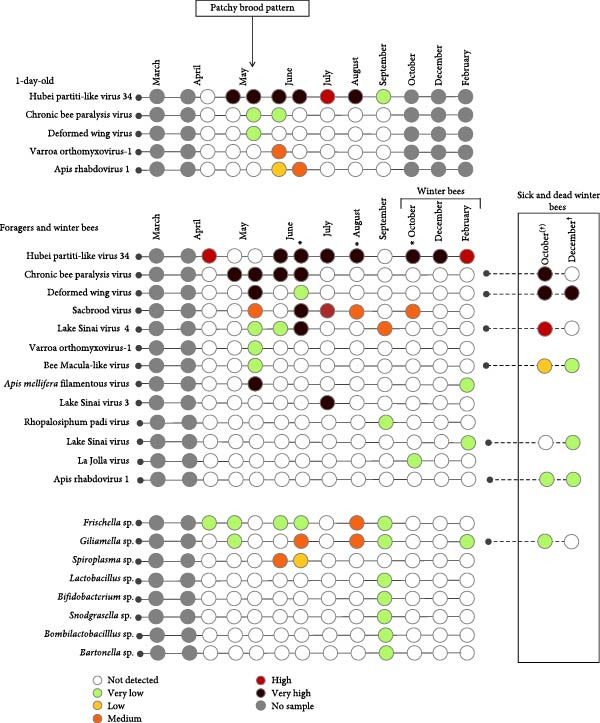
(C)

There was an age distribution of the pathogens. Five viruses were detected in 1‐day‐old bees: Apis rhabdovirus‐1, Chronic bee paralysis virus, Deformed wing virus, Hubei partiti‐like virus 34, and Varroa orthomyxovirus‐1. In foragers, 11 viruses were detected: *Apis mellifera* filamentous virus, Bee Macula‐like virus, Black queen cell virus, Chronic bee paralysis virus, Deformed wing virus, Hubei partiti‐like virus 34, Lake Sinai virus, Lake Sinai virus 3 and 4, Sacbrood virus, and Varroa orthomyxovirus‐1. Nine viral species were detected in winter bees: *Apis mellifera* filamentous virus, Apis rhabdovirus 5, Chronic bee paralysis virus, Deformed wing virus, Hubei partiti‐like virus 34, La Jolla virus, Lake Sinai virus, Sacbrood virus, and Varroa orthomyxovirus‐1. Deformed wing virus, Lake Sinai virus, Lake Sinai virus 4, Bee Macula‐like virus, Apis rhabdovirus‐1, and Chronic bee paralysis virus were detected in the sick and/or dead winter bees. Multiple viruses were only detected in foragers: Bee Macula‐like virus, Black queen cell virus, and Lake Sinai virus 4. One virus was detected in one age group, La Jolla virus in winter bees. *Apis mellifera* filamentous virus, Deformed wing virus, Hubei partiti‐like virus 34, and Varroa orthomyxovirus‐1 were detected in all ages. Most bacteria were detected in the foragers, except for *Frischella* sp. and *Gilliamella* sp. which were also detected in 1‐day‐old bees. It is important to note that despite our efforts to prevent contamination of hemolymph samples, a few pooled samples were found to contain pollen, as revealed by cytospin analysis (Figure 2 and Supporting Information 1: File 1). These contaminants were not detectable through visual inspection alone. The presence of pollen may result from surface contamination or possibly from gut content leakage. As this contamination could influence the sequencing results, data interpretation should be approached with caution.

### 3.3. Hemocyte Analysis by Flow Cytometry

The THCs per apiary can be found in Supporting Information 1: File 1. Cytospin analysis of the individual samples revealed pollen (one forager sample of apiary A, one forager and three winter bee samples in apiary B, and two forager and one winter bee sample(s) in apiary C) or suspected parasite contaminated samples (eight forager samples in apiary A, eight forager samples in apiary B, and five forager samples in apiary C) (Supporting Information 1: File 1). The pollen contaminated bee samples were removed before analysis. The analysis was run with and without the suspected parasite contaminated samples. When two samples are taken in 1 month, the first sample is labeled as Month1 and the second as Month2.

The one‐way ANOVA revealed that there is a statistically significant difference of THC between the age groups in all apiaries, namely for apiary A: (*F* = 194.927, *p* < 0.001), apiary B: (*F* = 219.328, *p* < 0.001), and apiary C: (*F* = 196.822, *p* < 0.001). An effect for hive was visible in apiary B (*F* = 6.382, *p* < 0.001) and apiary C (*F* = 3.103, *p* = 0.027) but not in apiary A (*p* = 0.083). Bonferroni’s test for multiple comparisons found that the mean value of lg(THC) was significantly different between 1‐day‐old bees and foragers (apiary A: *p* < 0.001, apiary B: *p* < 0.001, and apiary C: *p* < 0.001), 1‐day‐old bees and winter bees (apiary A: *p* < 0.001, apiary B: *p* < 0.001, and apiary C: *p* < 0.001), and foragers and winter bees (apiary A: *p* < 0.001 and apiary C *p* = 0.009). Not significant in apiary B for foragers compared to winter bees (*p* = 1.000). Removal of the suspected parasite contaminated samples yielded the same pattern of results, so they were kept in the dataset. In Apiary A, B, and C, no outliers were identified that could influence the results.

**Figure 3:**
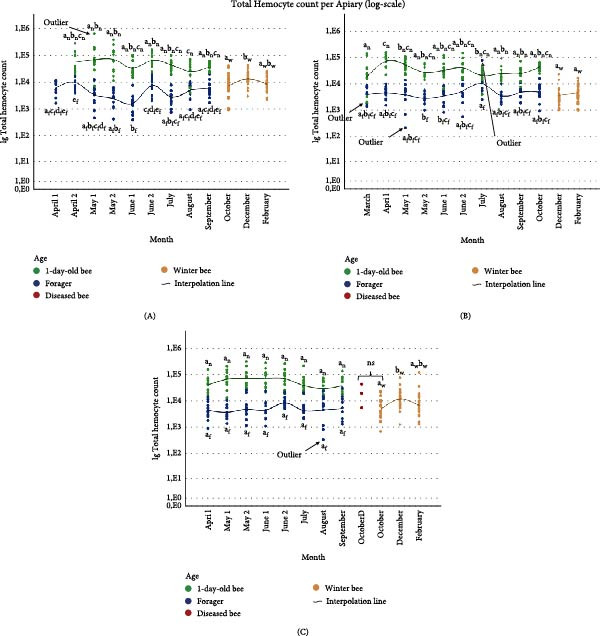
Scatterplot (log‐scale) of total hemocyte count by age group, with an interpolation line indicating the mean for each group. 1‐day‐old total hemocyte counts are indicated in green and subscript *n*, forager total hemocyte counts in blue and subscript *f*, winter bee total hemocyte counts in orange and subscript *w*, and total hemocyte counts of diseased winter bees (OctoberD) of apiary C in red and no subscript. Letters denote significant group differences within each analysis for the entire log‐transformed dataset (including outliers and potentially parasite infected samples) (Bonferroni, *p*  < 0.05). Different letters indicate significantly different means; shared letters or ns indicate nonsignificant differences. (A) Apiary A, (B) Apiary B, (C) Apiary C.

The effect of sampling date on THC depended on the apiary and the age of the bees. The results of the entire dataset including outliers and potentially parasitized samples are given in Figure 3. A significant effect of sampling date on lg(THC) was seen in every age group in apiary A (1‐day‐old *F* = 3.664, *p* = 0.002, foragers *F* = 6.823, *p* < 0.001, winter bees *F* = 3.579, *p* = 0.033). There was also an effect of hive on the THC in the 1‐day‐old bees (*F* = 11.656, *p* < 0.001) and the foragers (*F* = 2.826, *p* = 0.043) but not for the winter bees (*p* = 0.086). Bonferroni’s test for multiple comparisons of the 1‐day‐old bees found a significant difference (*p* < 0.05) between the results of August and June 2 and August and May 2. However, the August versus May 1 comparison included a notable outlier in group May 1 (*Z* score value = 3,11279) which may have affected the result. When this outlier was removed, the difference between May 1 and August became not significant (*p* = 0.133), the other comparisons yielded the same pattern of results. In the foragers, a significant effect (*p* < 0.05) of sampling date on THC was visible when we compared April and June 1, April 2 and July, April 2 and June 1, April 2 and May 1, April 2 and May 2, August and June 1, June 1 and June 2, June 1 and September, and June 2 and May 2. Removing suspected parasite contaminated samples from the analysis did not influence the results of the overall ANOVA, only the post hoc analysis of April 2 compared to May 1 became not significant (*p* = 0.077). The other results were comparable. No outliers were identified in either the dataset with or without potentially parasite contaminated samples. Bonferroni’s test for multiple comparisons of the winter bees found a difference between December and October (*p* = 0.041). No outliers were identified in the winter bees’ samples.

A significant effect of sampling date on THC was observed in both 1‐day‐old bees (*F* = 4.340, *p* < 0.001) and foragers (*F* = 2.383, *p* = 0.017) in apiary B. No statistically significant difference in THC was detected between winter bees sampled in December and February (*p* = 0.165). A significant hive effect on lg(THC) was found across all groups: 1‐day‐old bees (*F* = 5.101, *p* = 0.002), foragers (*F* = 7.047, *p* < 0.001), and winter bees (*F* = 10.905, *p* < 0.001). Bonferroni‐adjusted post hoc tests for the 1‐day‐old bees indicated significant pairwise differences between (*p* < 0.05) in April and August, April and July, April and March, April and September, and March and May 1. One outlier was identified in March (*Z* = −3.45607). Removal of this outlier altered the post hoc outcomes: the difference between March and May 1 became nonsignificant (*p* = 0.188), while a new significant difference emerged between May 2 and April (*p* = 0.047). In foragers, a significant effect of sampling date (*p* < 0.05) was reflected in pairwise comparisons between July and June 1 and July and May 2. Excluding samples suspected of parasite contamination did not meaningfully affect the results so they were kept in the dataset. Two outliers were identified: one in May 1 (*Z* = −3.58632) and one in July (*Z* = 3.35099). Removal of these outliers impacted the July and June 1 comparison making it not significant (*p* = 0.189), the other results were not meaningfully altered.

In apiary C, a significant effect of sampling date on THC was observed in 1‐day‐old bees (*F* = 2.785, *p* = 0.012) and winter bees (*F* = 5.741, *p* = 0.005), but not in foragers (*p* = 0.395). Conversely, a significant effect of hive was found in the foragers (*F* = 4.082, *p* = 0.009), but not in the 1‐day‐old bees (*p* = 0.315) or winter bees (*p* = 0.934). In the 1‐day‐old bees, post hoc comparisons did not identify any statistically significant pairwise differences between groups. No outliers were identified which could alter the results. Removing samples with suspected parasite contamination had no impact on the forager results, so all samples were retained. One outlier was identified in the foragers from August (*Z* = −3.14030), but its removal did not alter the findings. However, for winter bees, a significant difference was found between December and October (*p* = 0.004). No outliers were identified. When THC data was compared between the healthy and diseased October samples no significant differences were found (*p* = 0.978).

**Figure 4:**
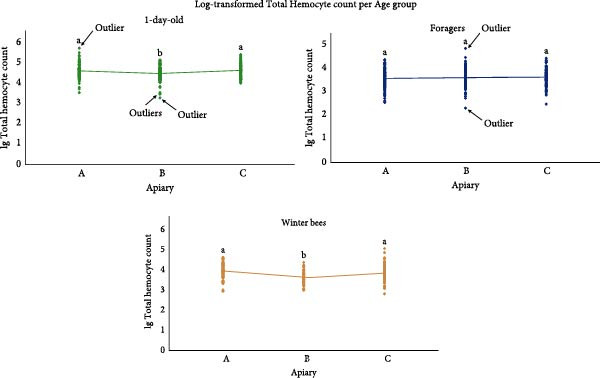
Scatterplot of lg transformed total hemocyte count per apiary for every age group (nurses, foragers, and winter bees). Letters denote significant group differences within each analysis for the entire dataset (including outliers and potentially parasite infected samples) (Bonferroni, *p*  < 0.05). Different letters indicate significantly different means and shared letters indicate nonsignificant differences.

Apiary had a significant effect on log‐transformed THC levels in both the 1‐day‐old bee group (*F* = 6.074, *p* = 0.003) and the winter bee group (*F* = 9.532, *p* < 0.001), but not in the forager group (*p* = 0.552). Post hoc comparisons (Bonferroni‐adjusted) revealed significant differences in THC counts between apiary A and B (1‐day‐old: *p* = 0.022; winter bees: *p* < 0.001), and between apiary C and B (1‐day‐old: *p* = 0.005; winter bees: *p* = 0.012). No significant differences were observed between apiary A and C for either group (1‐day‐old: *p* = 1.000; winter bees: *p* = 0.309), Figure 4. The removal of suspected parasitized samples did not alter the statistical outcomes; therefore, these samples were retained in the final dataset. Outlier analysis identified four extreme values in the 1‐day‐old group (apiary B: *Z* = –3.63213, *Z* = –3.09106, and *Z* = −3,10698; Apiary A: *Z* = 3.28362) and two in the forager group (apiary B: *Z* = –3.43171 and *Z* = 3.28311). Exclusion of these outliers notably affected the 1‐day‐old group results, rendering the comparison between apiary A and B non significant (*p* = 0.152). The overall ANOVA remained significant with or without the outliers (*F* = 4.101, *p* = 0.017).

### 3.4. *Nosema* sp. Analysis Using a Bürker Counting Chamber and qPCR

**Figure 5:**
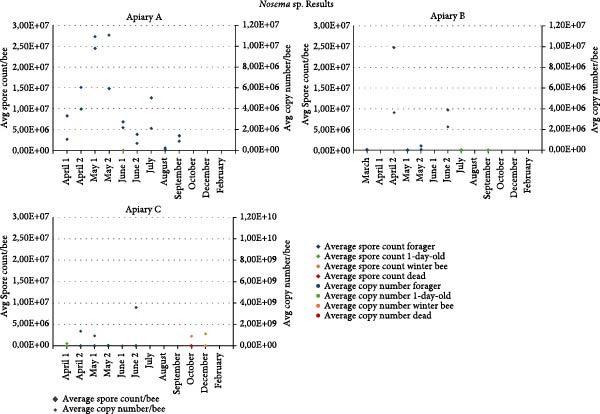
*Nosema* sp. counting (average spore count per bee, diamond) and qPCR results (average copy number per bee, circle) for every apiary. One‐day‐old bees are indicated in green, foragers in blue, winter bees in orange, and dead bees in red.

The results of the qPCR (*Nosema ceranae*) and Bürker counting chamber can be found in Figure 5. The detailed results can be found in Supporting Information 3: File 3. *Nosema* sp. loads varied based on beekeeper, age, and season. In general, *Nosema* sp. occurred at higher values in spring compared to summer and winter. *Nosema* sp. also occurs more frequently and at higher loads in foragers, compared to nurses. For the forager samples we can see two peaks, one in approximately May and one in June for all apiaries. The positive samples had counts up to 10^7^ spores per bee and copy numbers for *N. ceranae* in the up to 10^7^ copy numbers per bee. Apiary A had the highest *Nosema* sp. load, while apiary B and C had lower counts/loads.

### 3.5. *Varroa* Mite Counting

**Figure 6:**
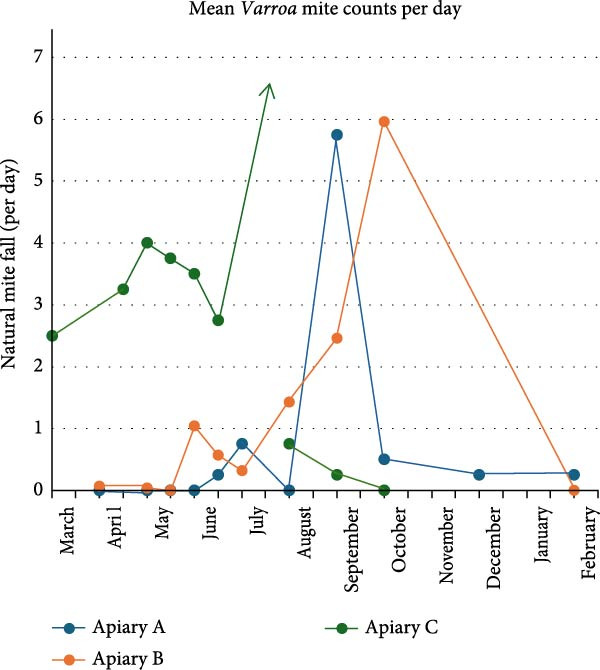
Mean *Varroa* mite counts per sampling. In July in apiary C, the natural mite fall was too high to count due to treatment, which is represented with an arrow. The tick marks on the horizontal axis represent the start and end of each month.

Figure 6 presents the mean natural mite fall per sampling. Apiaries A and B initially exhibited low natural mite fall, which increased during the bee season. In contrast, apiary C started with a higher natural mite fall that declined following oxalic acid treatment (July). In apiary A, the highest natural mite fall was present in September. In apiary B, the highest natural mite fall was observed in October. In apiary C, the natural mite fall was so high in July (after oxalic acid treatment) that counting was not performed.

## 4. Discussion

In this study, we used advanced sequencing technologies and flow cytometry to analyze pathogens and hemocytes in hemolymph samples from nurses, foragers, and winter bees in three apiaries throughout a year/honey bee season. Additionally, we examined the abdomen samples for *Nosema* sp. and tracked *Varroa* mite infestation levels.

Using third‐generation nanopore sequencing, we identified a diverse array of viruses and bacteria in honey bee hemolymph. This approach offers a distinct advantage over traditional methods that analyze whole bees, as it enables the detection of active infections by focusing on pathogens present in the circulatory system, thereby minimizing contamination from gut contents or surface microbes. However, it is important to note that comparing our findings with existing literature requires caution. Most studies utilize whole‐bee analyses, which can detect both active infections and residual or noninfectious genetic material, potentially leading to discrepancies when juxtaposed with hemolymph‐based results. Additionally, certain pathogens that localize primarily in the gut or other tissues may not be present in hemolymph samples, potentially leading to underrepresentation in our analysis [25]. Therefore, while our methodology provides a more targeted insight into systemic infections, direct comparisons with whole‐bee studies should be approached with an understanding of these methodological differences. A limitation of this study is that hemolymph sampling is more labor‐intensive than analyzing whole bees, which restricted the number of bees and colonies that could be included and increased the likelihood of missing infections. In addition, the use of third‐generation sequencing involves considerable costs, further constraining the number of samples analyzed.

Some identified viruses were never detected before in Belgian honey bees (such as Varroa orthomyxovirus‐1 and Hubei partiti‐like virus 34) or even in European honey bees (including La Jolla virus and Apis rhabdovirus 5) [26]. La Jolla virus usually infects *Drosophila* species, but it has been identified in *Apis* species in Australia and Ethiopia [26, 27]. Hubei partiti‐like virus 34 has recently been reported in Slovenia and has been reported in other continents [26, 28, 29]. It has been detected in two other species, namely in *Vespa velutina* (Italian isolate [MT747982] and Stylommatophora (Chinese isolate [KX884207]). In general, a broad range of viruses were identified. Especially in apiary C, which had the highest viral load of all three apiaries. Interestingly, this high viral diversity did not lead to noticeable reduced colony production in all apiaries as is consistent with previous findings [30]. Though, there was a patchy brood pattern in apiary C at the end of May. A patchy brood pattern is sometimes associated with bad queen productivity, but this has been debated [31]. Another cause for patchy brood can be diseased larvae or pupae that are removed from the combs as a form of hygienic behavior [32]. During this patchy brood period, different viruses were detected in the pooled samples of both 1‐day‐old bees and forager bees which could be correlated to this symptom. A probable cause of this irregular brood pattern is Sacbrood virus, which causes larval mortality. Hygienic colonies can remove dead larvae, leading to more empty cells [33]. Sacbrood virus was also detected after the symptoms were gone and in other apiaries without any symptoms, thus additional factors were at play to transform this infection from a covert infection to an overt infection [12, 34]. Other factors contributing to the disturbed brood pattern may include *Varroa* mites as such or as mechanical or biological vectors of viruses. *Varroa* mites parasitize pupae, facilitating the transmission of several viruses, resulting in mortality. Of the identified viruses, Deformed wing virus and Varroa orthomyxovirus‐1 are both confirmed or suggested to utilize *Varroa* mites in their transmission [35, 36]. Parasitizing of the mites themselves can also cause hygienic behavior of the bees with removal of pupae. Although, no higher *Varroa* mite fall was observed. Additionally, chronic bee paralysis virus is speculated to be vertically transmitted, potentially leading to pupal mortality [25, 37, 38].

Overall, the pathogen load varied per apiary and per age group with the lowest pathogen load in 1‐day‐old bees, which aligns with previous studies [13, 39, 40]. Interestingly, of the five viruses identified in 1‐day‐old bees, three have been indicated to utilize, among others, *Varroa* mites for their transmission. These viruses include Deformed wing virus, Varroa orthomyxovirus‐1, and Apis rhabdovirus‐1, among which Deformed wing virus is the sole virus definitively confirmed to employ *Varroa* mites for transmission [35, 36, 41]. Additional viruses associated with *Varroa* mites, such as Bee‐Macula like virus and La Jolla virus, were exclusively detected in foragers or winter bees. Bee‐Macula like virus, Varroa orthomyxovirus‐1, La Jolla virus, and Apis rhabdovirus‐1 are suggested to replicate in *Varroa* mites as negative‐sense RNA has been detected in these mites [26, 35, 41, 42]. In general, the *Varroa* associated viruses were mostly identified on the apiary which only treated the honey bees once chemically against *Varroa* mites compared to the more frequent treatments in the other apiaries, suggesting that a more conservative *Varroa* treatment approach can lead to higher amounts of *Varroa* associated viruses.

**Figure 7:**
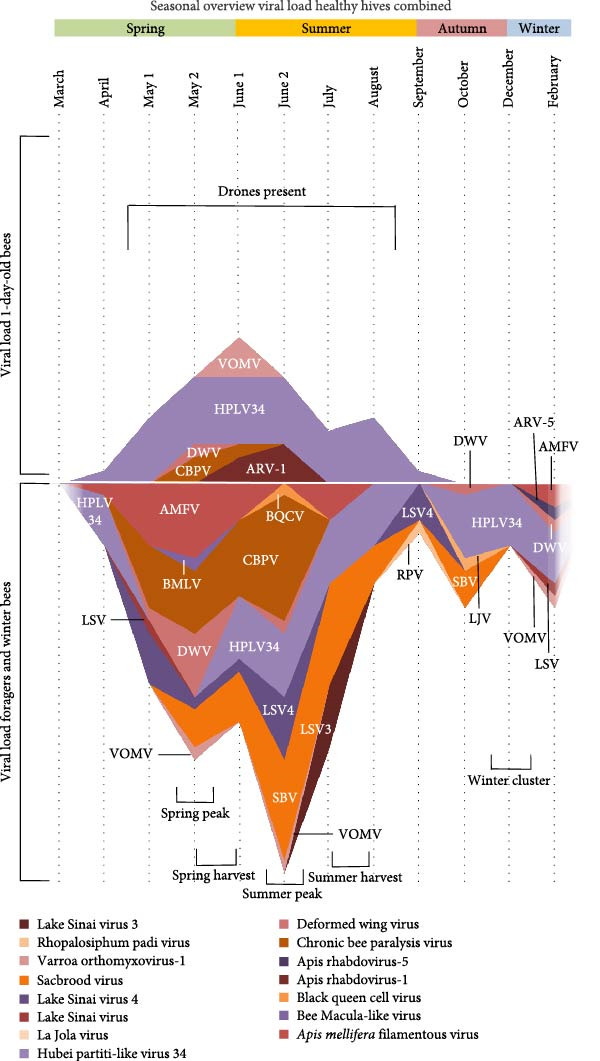
Seasonal overview of the viral load of the “healthy” hives combined, the results of the sick and dead honey bees are not included. The combined viral load of all individual viruses was tallied for each sampling across all apiaries, with ratings ranging from very low (1) to very high (5).

Most viruses were present in spring and summer (Figure 7). This “pathogen peak” was also observed in other studies but not always in the same period [13, 43–46]. The 1‐day‐old bees’ pathogen peak was highest in June, which coincides with the period in which the most brood is present [47]. In contrast, in foragers this peak was divided into two peaks. These two peaks coincided with the two flowering periods (spring and summer). In two studies, performed in the United Kingdom and Germany, the foraging distance was observed to be highest in summer and lowest in spring. This long flight distance in summer could explain why the summer peak is higher than the spring peak as bees can be exposed to a wider variety of pathogens in a bigger area [48, 49]. Notably, our study only observed a small peak in autumn. This is surprising as the foraging distance should be longer in autumn than in spring [48]. Moreover, the honey bee forager population reaches a peak at the start of July [47]. A higher number of individuals can increase viral transmission [50], but our observed pathogen loads do not follow this pattern. If we compare the results to our previous work, we notice that the pathogen peak is later (end of May instead of March/May) [13]. This can be explained due to the hotter and dryer climate of 2022 compared to 2023. The “bee season” started sooner with a pathogen peak that followed. So, it could be that variation in reported pathogen peak occurrence is partially due to climate differences or yearly fluctuations.

Additionally, after the viral infection peak, the viruses present in this peak, were either not detected again or the viral load was lower. This is surprising as there is a continuous presence of new bees and a continuous risk of reintroducing viruses and bacteria from other apiaries or other insects [37, 51, 52]. This pattern is reminiscent of transgenerational immunity, a process observed in honey bees that operates at the colony‐wide level [53]. The existence of transgenerational immunity in honey bees was first reported for the bacteria *Paenibacillus larvae*, which causes American foulbrood. Research on transgenerational immunity against viral infections in honey bees has mainly focused on a transmissible RNA pathway as honey bees defend themselves against viral infection via RNA interference for which dsRNA is the trigger. Additionally, transgenerational immunity could explain why viral loads stay low during autumn as the colonies have been primed during spring and summer.

Some viruses did not follow this pattern and were detected multiple times at high levels. These viruses were Hubei partiti‐like virus 34 in the healthy hives in apiary C and Chronic bee paralysis virus, Deformed wing virus, and Lake Sinai virus 4 in the sick hives of apiary C. Hubei partiti‐like virus 34 was endemic in apiary C and more interestingly also in 1‐day‐old bees, which usually have a low pathogen load [13, 39, 40]. This endemic infection, which continued into the winter bees, was remarkably not detected in the sick bees or dead bees of apiary C. This is in contrast to what was observed in the studies of Kwon [28] and Kramar [29], which both found Hubei partiti‐like virus 34 in diseased colonies. Because Hubei partiti‐like virus 34 was just recently discovered, not much is known about this virus. Kramar [29] suggested that its detection might be the result of surface contamination. The presence of Hubei partiti‐like virus 34 in honey bee hemolymph, its ability to evade the immune system, and its persistence over time are causes for concern. This infection can be persistently covert, which can lead to weakening of the honey bees as seen in covert Deformed wing virus infections, or this infection can be chronically overt with minimal symptoms, which have not been described yet [10–12, 54].

All viruses detected in the sick bees had been detected previously on this apiary. This implies that the affected hives were unable to eradicate the viruses upon their initial/reintroduction or maintain the viruses at a minimal level compared to the healthy hives. An example is a covert infection reemerging as an overt infection, previously established for different viruses some of which were identified in the sick hives such as Deformed wing virus and Chronic bee paralysis virus [37, 55, 56]. This can explain why these viruses did not follow the previously established pathogen peaks. A reason for the high viral loads is an immune system collapse. This collapse could be due to the conservative *Varroa* treatment approach as three out of four identified viruses are associated with *Varroa* mites [35, 36, 41, 42]. Though, this oxalic acid treatment can also induce honey bee mortality [57, 58]. Our results also suggest this as the dead bees in apiary B after oxalic acid treatment did not contain high pathogen loads. Apiary C had a high colony density compared to the other apiaries. High colony density has been correlated to higher viral prevalence [59]. There was no high bacterial load present in the sick or dead bees suggesting that bacterial infections were less important in the downfall of the hives. In previous studies Chronic bee paralysis virus, Bee‐Macula like virus, Deformed wing virus, and Lake Sinai virus 4 were also identified in dead/sick colonies [13, 28, 60–62]. Additionally, some other detected viruses have been identified in dead overwintering colonies such as Lake Sinai virus 3 and Sacbrood virus [28].

The sick and dead hives were present on apiary C, which was the apiary with the highest pathogen load. The total amount of viruses and the viral load of the sick bees was higher than that of the healthy bees in the same sampling period (apiary C). This is in line with previous studies [28, 63, 64]. Interestingly, the viral load in the sick bees and dead bees (apiary C) was similar but not identical. In the dead bees, the viral load was lower and Chronic bee paralysis virus was not detected. As it is difficult to determine the exact time of death, the dead bees could have been dead months before the samples were taken. Chronic bee paralysis virus is an enveloped virus, which is less stable compared to nonenveloped viruses such as Deformed wing virus. It is possible that Chronic bee paralysis virus broke down before the samples were collected [65]. Lake Sinai virus and Bee Macula‐like virus loads were also reduced but it is unknown if they are enveloped [66]. Another possibility is that the dead bees were not all infected before they succumbed due to cold as the low number of bees cannot maintain a sustainable temperature. This stresses the importance of sampling sick individuals instead of dead bees. But this is not an easy task as disease symptoms can be subtle, as observed in this instance. The sick bees were observed motionless at hive entrances, albeit in small numbers. Since hive entrances typically remain devoid of dead bees, this was deemed as “abnormal.” Familiarity with typical hive conditions and honey bee behavior is essential for identifying such subtle cues. It is also possible that the Chronic bee paralysis virus infection was missed due to this small amount of life sick bees.

Bacterial loads also occurred in a seasonal pattern with the highest load present in spring in foragers, as has been observed before [13, 39]. Apiary B had the highest bacterial loads with *Spiroplasma* sp. reaching high values and *Arsenophonus* sp. reaching very high levels. In the sick and dead bees, bacteria did not reach high levels, as has been noted before [13]. *Spiroplasma* sp. are a known honey bee pathogen, but *Arsenophonus* sp. are not [67].

*Nosema sp*. also occurred at higher values in spring compared to summer and winter, which has been observed before [68–70]. Although it was generally accepted that only *Nosema apis* followed a seasonal pattern and that *Nosema ceranae* could be found all year round [71]. As we saw previously for viral and bacterial presence, *Nosema* also occurs more frequently and at higher loads in foragers, compared to 1‐day old bees, which has been observed before [40]. Additionally, the sick winter bees in apiary C were negative for *Nosema* sp. The dead bees were infected but so were the winter bees. *Nosema* sp. replicates in the epithelial cells of honey bees, causing severe tissue damage. This damage could be an important entry portal for other pathogens [6]. Interestingly, the apiaries with the highest and most diverse viral (apiary C) and bacterial (apiary B) loads had the lowest *Nosema* counts.

Research on THCs in adult insects in general is scarce. THCs in honey bees varied based on age, season, and apiary. First, the lg(THC) of foragers was significantly lower compared to 1‐day‐old bees in all apiaries. A depletion of hemocyte counts as adult honey bees age, and by extension other insects, has been reported before but contradictory information exists [39, 72–75]. Therefore, we conducted a cage trial to analyze hemocyte levels in aging honey bees in absence of pathogen pressure and confirmed their depletion [76]. A significant difference was also observed between winter bees and 1‐day‐old bees or foragers bees in apiary A and C. This is not surprising, given winter bees differ immunologically from young bees and forager bees [77]. Interestingly, this difference between winter bees and foragers was not visible in apiary B. This could be due to, for example, the smaller sample size of the winter bees in apiary B. Contradictory, in the research of Kostecki [78], winter bee hemocyte counts were lower than 1‐day‐old bees and 30‐day old bees but similar to 50‐day old bees [78]. Second, there was a significant effect of sampling date. In the winter bees a significant difference is observed between the results of October and December in both apiary A and C and no difference between December and February in all apiaries, Figure 3. It could be that the October sample did not purely contain winter bees but also some forager bees with lower THC. These forager bees normally die within 4 weeks so in December only pure winter bees should be left. Remarkably, an effect of sampling date was also visible in the 1‐day‐old bees in apiary A and B and the foragers in apiary A and B, Figure 3. To our knowledge this is the first study on honey bee and even insect THCs during the year. Given the numerous factors that can influence hemocyte profiles, pinpointing the specific factors at play remains challenging [79, 80]. As hemocytes play a crucial role in combatting diseases, understanding why this effect of sampling date occurs, especially as this effect was most clear in the apiaries with a minimal viral and bacterial load, can be vital to understanding honey bee disease and mortality [79]. Surprisingly, this effect of sampling date was absent in apiary C, where 1‐day‐old bees and foragers showed higher rates of viral infections. These multiple infections may lead to consistently elevated hemocyte levels [75, 81]. Third, although the removal of outliers altered the post hoc analysis, the overall effect stayed the same namely an effect of apiary on 1‐day‐old lg(THC) and winter bee lg(THC) counts. The fact that immune parameters are influenced by location is not unsurprising, as for example pesticides and food resources can influence hemocyte parameters [82, 83]. Additionally, different management strategies and the different genetic backgrounds of the bees can also influence immune parameters or survival [84–86]. Surprisingly, this effect of apiary on lg(THC) was not visible for the foragers. As stated previously, hemocytes are understudied so explaining the results is challenging. The goal of this study was to give a descriptive overview of the possible effects of several parameters on hemocyte counts. In the future more research should be done to understand hemocyte dynamics and care should be taken when hemocytes are studied as sampling date can have an effect. Our related study, which monitored hemocyte dynamics in aging honey bees under controlled cage conditions, provides a baseline for healthy hemocyte trends over time. This observed depletion warrants further investigation across different honey bee breeds, seasonal contexts, and varying pathogen pressures [76].

The flushing techniques has advantages as a high amount of cells can be retrieved per bee compared to alternative techniques especially for forager bees who are difficult to collect hemolymph from [87]. Although our technique is promising, there is a higher risk of contamination as the pressure of the flushing fluid in the bee can cause a defecation response or even a prolapse of the rectum. If the puncture wound is made too big, the intestine can protrude out of the puncture hole which results in a risk of contamination. This contamination was seen in a small number of samples. As the contamination was microscopic, this contamination was deemed minimal, especially as it was diluted in the pooled samples. For example, in the December winter bees of apiary B, two contaminated bees were included in the pooled sample, but no bacteria or viruses were detected. All viruses and bacteria detected in the contaminated pooled samples were also detected in the noncontaminated samples so we deemed the effect of this contamination minimal. Additionally, some parasitic like structures were identified in the cytospins namely *Nosema* like structures and Trypanosoma‐like structures. *Nosema ceranae* genetic material has been identified in honey bee hemolymph before [88]. Trypanosoma’s, namely *Crithidia* sp. and *Lotmaria passim* have been identified in honey bees [89, 90]. The latter both in honey bee adults and the brood [91, 92]. They attach and replicate in the hindgut of honey bees [93]. A presence in the hemolymph has not been described, although *Crithidia* sp. are capable of multiplying in the hemolymph after injection in several insect species [94]. Additionally, related monoxenous species have been identified in the hemolymph, although it is rare [95].

In conclusion, this study monitored honey bee pathogens and THCs over the course of a single honey bee season. As our per‐apiary sample size was constrained by the labor and costs of hemolymph extraction and third‐generation sequencing, we chose an exploratory approach while acknowledging that this reduces detection power. Despite these limitations, this study provides a broad overview of pathogen presence in apiaries and has generated several new hypotheses and observations. Future research by our group will build on these findings, with larger sample sizes and more efficient use of resources to test these hypotheses more comprehensively. Our findings revealed a potential seasonal pattern for pathogens and an effect of sampling date on THCs, with the latter being reported for the first time. Additionally, some identified viruses were never detected before in Belgian honey bees (such as Varroa orthomyxovirus‐1 and Hubei partiti‐like virus 34) or even in European honey bees (including La Jolla virus and Apis rhabdovirus 5). Understanding these patterns in healthy hives provides a baseline that can aid in the identification of diseased hives and contribute to a deeper understanding of factors influencing colony mortality.

## Disclosure

The funders had no role in the study design, data collection, data interpretation, or decision to submit the work for publication.

## Conflicts of Interest

Sebastiaan Theuns and Nauwynck Hans are cofounders and co‐owners of PathoSense BV. Sieglinde Coppens is an employe at PathoSense BV. The other authors declare no conflict of interest.

## Author Contributions

Dirk C. de Graaf and Hans Nauwynck contributed equally to this work.

## Funding

This work was supported by the Research Foundation–Flanders (FWO) (Grant Number 1SB3123N).

## Supporting Information

Additional supporting information can be found online in the Supporting Information section.

## Supporting information


**Supporting Information 1** File 1: This supporting file includes the detailed hemocyte counts, cytospin remarks, and sampling dates.

**Supporting Information 2** File 2: This supporting file contains an overview of the viral genomes and their GenBank accession codes. Due to the error rate of nanopore data, erroneous insertions or deletions may persist within genomes, particularly in regions characterized by low coverage, homopolymers, and repeats. A final manual curation is performed to ensure these are removed. The genome is aligned to the closest NCBI reference, and all inserts and deletions are checked and corrected if necessary.

**Supporting Information 3** File 3: This supporting information file contains the detailed *Nosema* sp. counts and qPCR results.

## Data Availability

The data that supports the findings of this study are available in the supporting information of this article.
